# ATP-Evoked Intracellular Ca^2+^ Responses in M-CSF Differentiated Human Monocyte-Derived Macrophage are Mediated by P2X4 and P2Y11 Receptor Activation

**DOI:** 10.3390/ijms20205113

**Published:** 2019-10-15

**Authors:** Janice A. Layhadi, Samuel J. Fountain

**Affiliations:** Biomedical Research Centre, School of Biological Sciences, University of East Anglia, Norwich Research Park, Norwich NR4 7TJ, UK; janice.lee-layhadi07@imperial.ac.uk

**Keywords:** macrophage, calcium signaling, purinergic, inflammation

## Abstract

Tissues differentially secrete multiple colony stimulating factors under conditions of homeostasis and inflammation, orientating recruited circulating monocytes to differentiate to macrophage with differing functional phenotypes. Here, we investigated ATP-evoked intracellular Ca^2+^ responses in human macrophage differentiated with macrophage colony-stimulating factor (M-CSF). Extracellular ATP evoked (EC50 13.3 ± 1.4 μM) robust biphasic intracellular Ca^2+^ responses that showed a dependency on both metabotropic (P2Y) and ionotropic (P2X) receptors. qRT-PCR and immunocytochemistry revealed the expression of P2Y1, P2Y2, P2Y6, P2Y11, P2Y13, P2X1, P2X4, P2X5, and P2X7. Pharmacological analysis revealed contribution of only P2X4 and P2Y11 to the Ca^2+^ response evoked by maximal ATP concentrations (100 µM). This study reveals the contribution of P2X4 and P2Y11 receptor activation to ATP-evoked intracellular Ca^2+^ responses, and makes comparison with macrophage differentiated using granulocyte colony-stimulating factor (GM-CSF).

## 1. Introduction

The generation of monocyte-derived macrophage can be achieved by exposure to colony-stimulating factors (CSFs). CSF are secreted glycoproteins which can recognize and bind to receptors expressed on the surface of haemopoietic stem cells and stimulate proliferation and differentiation. CSFs are classically characterised in three types, as follows: *CSF1* – macrophage colony-stimulating factor (M-CSF); *CSF2* – granulocyte macrophage colony-stimulating factor (GM-CSF); and *CSF3* – granulocyte colony-stimulating factor (G-CSF). While M-CSF is expressed in abundance by many tissues during homeostasis [1,2], GM-CSF is found at very low basal levels but significantly elevated in response to inflammation [3]. M-CSF and GM-CSF activate different receptors, but both are capable of promoting macrophage survival, proliferation, differentiation, and activation [4,5]. M-CSF orientated human blood monocytes are widely used to generate monocyte-derived macrophage and model tissue macrophage. Macrophage differentiated using M-CSF are often referred to as M2-macrophage (alternatively activated) with an anti-inflammatory cytokine profile [6]. M2-macrophage can also be induced by pathogens, interleukin (IL)-4, IL-10, IL-13, and transforming growth factor (TGF-β) [7,8]. M2-macrophage were first described by Akagawa et al. as cells resembling peritoneal macrophage [9]. Conversely, macrophage differentiated using GM-CSF have a pro-inflammatory cytokine profile, referred to as M1-macrophage (classically activated), and resemble tissue macrophage in lung alveoli [9]. M1-macrophage can also be induced by IL-1β, TNF, IL-12, IL-18, and IL-23 [10]. Hence, circulating monocytes that extravasate into tissues expressing different CSFs can differentiate into macrophage with differing properties. The functional differences between these two types of macrophage in human tissues remain unclear though M1-polarised macrophage are generally associated with inflammation and M2-polarised are associated with anti-inflammatory effects. Reversibility between these polarised states has been observed [11].

In leukocytes, adenosine 5′-triphosphate (ATP) is a danger-associated molecular pattern (DAMP), released by damaged and inflamed tissues. In addition, ATP is physiologically released by healthy cells in response to stimulation, including mechanical distortion, hypoxia, and others [12]. The biological effects of ATP are mediated by activation of metabotropic (P2Y) and ionotropic (P2X) purinergic receptors. Purinergic receptors have received much attention as a route to the pharmacological manipulation of macrophage function in inflammation [13]. Despite this, the functional repertoire of purinergic receptors in human macrophage orientated by differing CSFs remains elusive. This study focuses on the role of purinergic receptors in ATP-evoked intracellular Ca^2+^ responses in M-CSF differentiated human monocyte-derived macrophage.

## 2. Results

### 2.1. ATP Evokes Intracellular Ca^2+^ Responses in M-CSF Differentiated Macrophage

**Figure 1:**
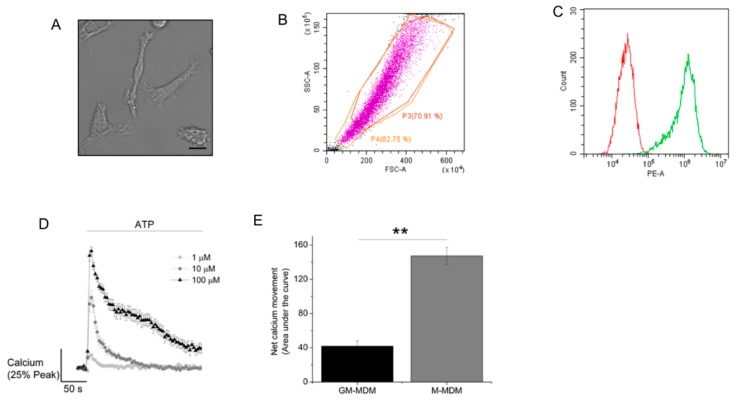
ATP evokes intracellular Ca^2+^ responses in M-CSF differentiated human macrophage. (**A**) Representative image showing macrophage differentiated from monocytes by M-CSF treatment (10 ng/mL, 6 days). Scale bar is 10 µM. (**B**) Flow cytometry forward and side scatter analysis. (**C**) Flow cytometry analysis of cell surface anti-CD14 immunoreactivity. Green channel for cells stained with anti-CD14 and red channel for antibody isotype control. (**D**) Averaged (3 donors) intracellular Ca^2+^ responses evoked by ATP at different concentrations. (**E**) Comparison of the total Ca^2+^ response evoked by 100 μM ATP in GM-CSF (10 ng/mL, 6 days) versus M-CSF differentiated macrophage (3 donors each). ** *p* < 0.01. All data is mean ± SEM; hypothesis testing by *t*-test.

Human macrophage produced by M-CSF differentiation of monocytes displayed phenotypically elongated characteristics and a highly vacuolated appearance [14] (Figure 1A), distinct from those differentiated with GM-CSF [15]. The appearance of this phenotype was further confirmed by forward and side scatter plot analysis via flow cytometer (Figure 1B). Flow cytometry analysis of anti-CD14 immunoreactivity revealed that approximately 97% of M-CSF differentiated cells were CD14 positive (Figure 1C), which is significantly higher than GM-CSF differentiated human macrophage [15]. ATP evoked an intracellular Ca^2+^ response that increased in magnitude with ATP concentration (Figure 1D). Responses at 10 and 100 μM ATP displayed a rapid rising phase and return to baseline. The size of Ca^2+^ response (area under the curve) at maximal ATP concentrations were significantly larger at those observed previously for GM-CSF differentiated macrophage ([15]; Figure 1E).

**Figure 2:**
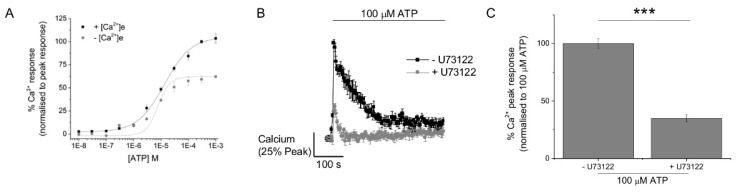
ATP evokes metabotropic and ionotropic Ca^2+^ responses in M-CSF differentiated human macrophage. (**A**) Averaged (*N* = 3) ATP concentration response performed in the presence (1.2 mM) and absence (no Ca^2+^, 2 mM EGTA) extracellular Ca^2+^. (**B**) Averaged (*N* = 3) intracellular peak Ca^2+^ responses evoked by 100 μM ATP in the absence and presence of U73122 (10 μM). Mean peak responses are given in (**C**) *** *p* < 0.01. All data is mean ± SEM; hypothesis testing by *t*-test.

ATP evoked Ca^2+^ responses with an EC_50_ value of 13.3 ± 1.4 μM (*N* = 3 donors) (Figure 2A). ATP was still able to elicit significant Ca^2+^ responses in the absence of extracellular Ca^2+^ (Figure 2A) and with a similar apparent potency (EC_50_ 7.8 ± 2.9 μM; *N* = 3 donors). Furthermore, phospholipase C inhibition with U73122 significantly reduced ATP-evoked Ca^2+^ responses (Figure 2B), though approximately 25% of the response was resistant to U73122 inhibition (Figure 2C). Taken together these data suggest that ATP-evoked Ca^2+^ responses in M-CSF differentiated human macrophage result from the combined activation of metabotropic (P2Y) and ionotropic (P2X) receptors for extracellular ATP.

### 2.2. M-CSF Differentiated Macrophage Express Multiple P2Y and P2X Receptor Subtypes

**Figure 3:**
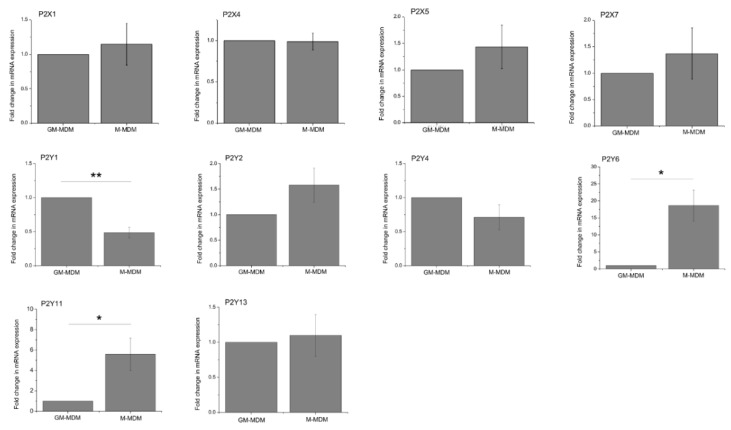
Comparative expression of P2Y and P2X receptor mRNA transcripts in GM-CSF versus M-CSF differentiated human macrophage. Quantitative RT-PCR analysis of mRNA transcripts (*N* = 5 donors). Transcript abundance is normalized to a *RPLP0* housekeeper gene as an internal control, and each transcript normalized to expression in GM-CSF differentiated macrophage for comparison of fold-change differences in mRNA abundance. * *p* < 0.05; ** *p* < 0.01. All data is mean ± SEM; hypothesis testing by *t*-test.

**Figure 4:**
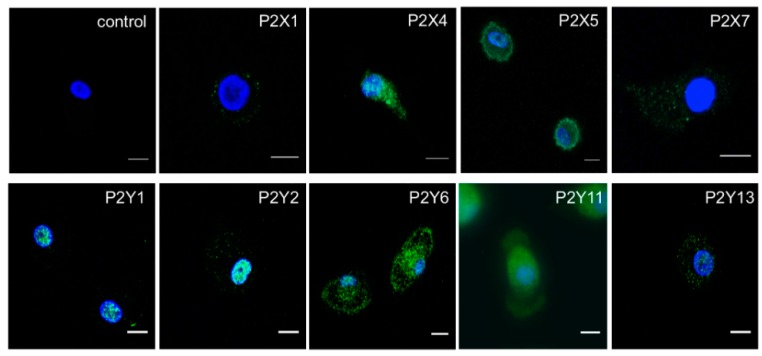
Immunocytochemistry of P2Y and P2X receptor expression in M-CSF differentiated human macrophage. Dual fluorescence images showing staining arising from the use of primary antibodies against receptor subtypes in green and nucleus counterstain in blue (DAPI). Control is representative of experiments performed in the absence of primary antibodies. Images representative of experiments performed on macrophage isolated from three independent donors. Scale bar is 10 μm.

To investigate the molecular basis for ATP-evoked Ca^2+^ responses, we first determined the expression of P2X and P2Y receptor subtype mRNA transcripts by quantitative RT-PCR. For P2Y receptors we explored both ATP and ADP activated receptor subtypes, as ADP can be liberated by the cell surface catabolism of ATP in macrophage [16]. Of the transcripts investigated for P2X receptors, P2X1, P2X4, P2X5, and P2X6 were expressed by M-CSF differentiated macrophage, and for P2Y receptors, P2Y1, P2Y2, P2Y4, P2Y6, P2Y11, and P2Y13 were detected. Those which were not detected in macrophage were detectable in cDNA isolated from human brain (data not shown). Further RT-PCR analysis of P2X5 revealed that all donors expressed the exon 10-less variant, and therefore express a non-functional P2X5 homomeric receptor [17] (data not shown). A quantitative comparison of mRNA expressed by GM-CSF versus M-CSF differentiated macrophage revealed comparable expression of P2X subtypes, and P2Y2, P2Y4, and P2Y13 receptors (Figure 3). P2Y1 was expressed in significantly less abundance by the M-CSF differentiated macrophage compared to the GM-CSF differentiated macrophage, though P2Y6 and P2Y11 were expressed in significantly greater abundance (Figure 3). The expression of P2Y and P2X receptor subtypes detected at the mRNA level were investigated further at the protein level by fluorescence immunocytochemistry (Figure 4). In these experiments, P2X1, P2X4, P2X5, P2X7, P2Y1, P2Y2, P2Y6, P2Y11, and P2Y13 showed clear expression in macrophage (Figure 4). P2Y4 was not investigated further due to the lack of a commercially available validated antibody.

### 2.3. P2Y11 and P2X4 Mediate ATP-Evoked Ca^2+^ Responses

**Figure 5:**
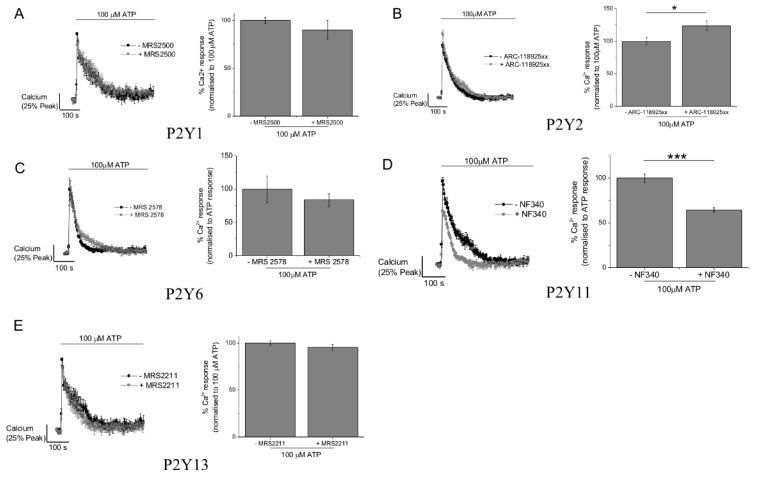
Effect of antagonizing P2Y receptor subtypes on ATP-evoked Ca^2+^ responses in M-CSF differentiated macrophage. For all panels (**A**–**E**), average Ca^2+^ response traces (*N* = 3) are shown to the left, with and without antagonist, and quantitated peak Ca^2+^ responses are shown in the bar chart to the right. Effects of receptor antagonism are shown for (**A**) P2Y1 (MRS2500, 1 μM), (**B**) P2Y2 (ARC-118925XX, 10 μM), (**C**) P2Y6 (MRS2578, 10 μM), (**D**) P2Y11 (NF340, 10 μM), and (**E**) P2Y13 (MRS2211, 10 μM). * *p* < 0.05; *** *p* < 0.01. All data is mean ± SEM; hypothesis testing by *t*-test.

**Figure 6:**
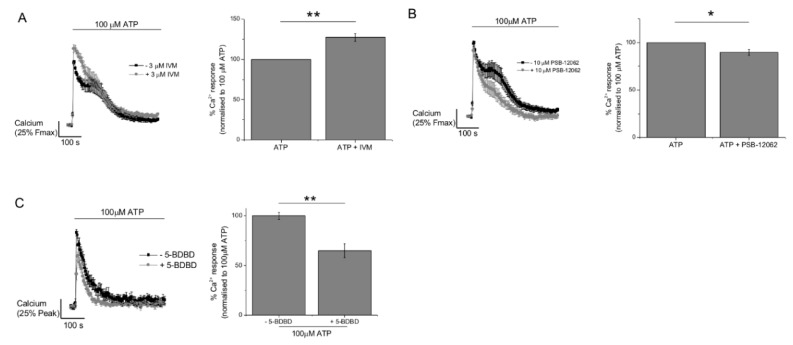
Contribution of P2X4 to ATP-evoked Ca^2+^ responses in M-CSF differentiated macrophage. For all panels (**A**–**C**), average Ca^2+^ response traces (*N* = 3) are shown to the left, with and without modulator/antagonist, and quantitated peak Ca^2+^ responses are shown in the bar chart to the right. Effect of (**A**) P2X4 positive allosteric modulator ivermectin (3 μM) and antagonists (**B**) PSB-12062 (10 μM) and (**C**) 5-BDBD (10 μM). * *p* < 0.05; ** *p* < 0.01. All data is mean ± SEM; hypothesis testing by *t*-test.

**Figure 7:**
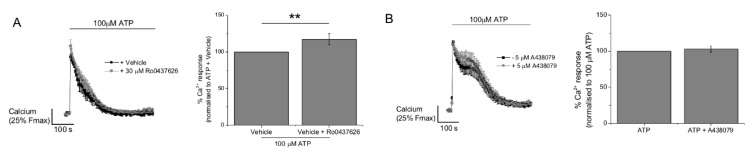
Lack of inhibitory effect of P2X1 and P2X7 receptor antagonists on responses activated by maximal ATP concentrations. For panels **A** and **B**, average Ca^2+^ response traces (*N* = 3) are shown to the left, with and without modulator/antagonist, and quantitated peak Ca^2+^ responses are shown in the bar chart to the right. Effect of (**A**) P2X1 receptor antagonism (Ro0437626, 30 μM) and (**B**) P2X7 receptor antagonism (A438079, 5 μM). ** *p* < 0.01. All data is mean ± SEM; hypothesis testing by *t*-test.

We next investigated the role of those receptor subtypes identified by immunocytochemistry using selective receptor antagonists and previously validated concentrations [15,18,19,20,21]. Antagonism of P2Y1, P2Y6, or P2Y13 had no significant effect of the ATP-evoked Ca^2+^ response (Figure 5). Antagonism of P2Y2 caused a minor but significant increase in the peak of the Ca^2+^ response, and P2Y11 receptor antagonism significantly inhibited the response (35.5 ± 2.6%, *N* = 3) (Figure 5). This data suggests that the Ca^2+^ response to ATP is partially mediated via P2Y11 activation. We investigated P2X1, P2X4, and P2X7 as potential candidates to mediate the ionotropic response to ATP. Ivermectin, a positive allosteric modulator of the P2X4 receptor, significantly increased (127.1 ± 5.0%, *N* = 7) the ATP-evoked Ca^2+^ response (Figure 6A). Conversely, PSB-12062 and 5-BDBD, two structurally unrelated P2X4 receptor antagonists, significantly inhibited the ATP-evoked Ca^2+^ response (Figure 6B,C). PSB-12062 inhibited the response by 10.5 ± 3.2% (*N* = 7) compared to 31.8 ± 9% (*N* = 4) for 5-BDBD. Both PSB-12062 and 5-BDBD inhibited the ivermectin potentiated response by 11.5 ± 4.5% (*N* = 6) and 16.3 ± 3.5% (*N* = 4), respectively. Interestingly, P2X1 antagonism with Ro0437626 caused a minor but significant increase in the response (Figure 7A). In keeping with our previous work on human primary macrophage [15], we observe that the P2X7 receptor antagonism had no effect on the maximal ATP-evoked Ca^2+^ response. Our interpretation of the ATP-evoked Ca^2+^ response pharmacology in human M-CSF differentiated macrophage suggests that P2Y11 and P2X4 contribute to receptor activation.

## 3. Discussion

Monocyte-derived macrophage differentiated using GM-CSF or M-CSF serve as a well-established tool to study primary tissue-specific macrophages [22]. In this study we observed that M-CSF and GM-CSF orientated human macrophage exhibit different purinergic responses and there are also differences in the molecular basis for this response. In M-CSF differentiated macrophage, ATP evoked a monophasic intracellular Ca^2+^ response consisting of a rapid peak and return to baseline. ATP evokes intracellular Ca^2+^ responses in M-CSF and GM-CSF differentiated macrophage [15] with similar potency (11–13 μM), though the net movement of Ca^2+^ in M-CSF differentiated macrophage was approximately 3-fold greater in M-CSF differentiated macrophage. As with GM-CSF macrophage [15] and model PMA-differentiated THP-1 cell macrophage [20] the majority of the magnitude of the ATP-evoked Ca^2+^response was due to activation of metabotropic P2Y receptors, revealed by U73122 sensitivity. Though not tested here, coupling of Ca2+ mobilisation to Orai1/STIM proteins are likely to be involved as well, as they have been shown to be important in GPCR-mediated Ca2+ signals in macrophage and microglia [23,24]. P2Y11 is a common contributor in ATP evoked responses in both M-CSF and GM-CSF differentiated macrophage, but P2Y13, despite contributing approximately 50% of the ATP evoked Ca^2+^ response in GM-CSF differentiated macrophage [15], does not contribute to the response in M-CSF macrophage. This is despite common expression in both macrophage types and the same mRNA abundance. The data suggest the functional contribution of P2Y13 can be used to discriminate between macrophage types. Experiments in human THP-1 monocytes suggest P2Y11 may be important in autocrine macrophage activation [25], however the cellular roles of P2Y13 are more elusive [26]. ADP is a full agonist at P2Y13 whilst ATP itself is a weak partial agonist [27]. Differences in the contribution of P2Y13 to ATP evoked responses in GM-CSF versus M-CSF could therefore be due to differences in the cell surface metabolism of ATP and the metabolic liberation of the full agonist ADP, though this requires further investigation.

A comparison of M-CSF differentiated macrophage in this study and GM-CSF differentiated macrophage in our previous work [15] suggests the ionotropic component of the ATP-evoked intracellular Ca^2+^ responses is greater in M-CSF differentiated macrophage than that observed in GM-CSF differentiated macrophage, based upon U73122 sensitivity. These findings suggest that monocytes that differentiate to macrophage in a M-CSF containing environment are equally sensitive to ATP as GM-CSF differentiated macrophage, but respond with a greater intracellular Ca^2+^ response, and P2X receptors contribute more to this response. In keeping with our previous observations in human primary macrophage [15], P2X7 does not contribute to the Ca^2+^ response at maximal ATP contributions. Others have observed P2X7 receptor contribution with the agonist BzATP, or at supramaximal ATP concentrations in the presence of unphysiologically low divalent cation concentrations [28,29,30]. A functional role of P2X1 is not supported here, and corroborates studies in human alveolar macrophage [28].

Monocyte-derived macrophages differentiated with colony stimulating factors GM-CSF or M-CSF are good in vitro models for the study of primary human macrophage [4]. Though the literature has become more polarized of late, GM-CSF differentiation and M-CSF differentiation are often associated with pro-inflammation and anti-inflammation, respectively, though this is context dependent. To this end, the macrophages used in this study would best model an anti-inflammatory or homeostatic scenario. The P2X4 receptor is of particular interest as its pharmacology has recently improved [31]. Based upon our pharmacological characterization, the contribution of P2X4 to the ATP-evoked Ca^2+^ responses is greater in GM-CSF compared to M-CSF macrophage. In GM-CSF differentiated macrophage, activation of the P2X4 receptor elicits secretion of the pro-inflammatory chemokine CXCL5 [15]. P2X4 antagonism in both macrophage types will therefore attenuate ATP evoked Ca2+ signaling. The cellular roles of P2Y11 and P2X4 in M-CSF differentiated macrophage are unclear and require further investigation.

In summary, our data reveal that human macrophage differentiated from monocytes exposed to different colony stimulating factors both respond to ATP by generating intracellular Ca^2+^ responses, though the molecular basis for this differs. In M-CSF differentiated macrophage, both P2X and P2Y receptors contribute to the response with P2X4 and P2Y11 receptors playing a dominant role. This study provides novel information regarding the molecular basis of ATP-evoked Ca^2+^ signals in human primary macrophage differentiated by M-CSF.

## 4. Materials and Methods

### 4.1. Peripheral Blood Mononuclear Cells (PBMC) Isolation

Human whole blood was obtained from healthy volunteers through the Faculty of Medicine and Health Sciences Research Ethics Committee (reference 2012/2013-03HT; approved 2012), University of East Anglia. Whole blood was centrifuged at 1000× *g* for 10 min to isolate plasma. Whole blood was diluted with RPMI medium in a 1:1 ratio and layered onto a Histopaque-1077 (Sigma Aldrich, St Louis, Missouri, USA) density gradient before centrifuging at 1000× *g* for 25 min. Mononuclear cells were collected by gently transferring the opaque interface into a fresh centrifuge tube. Cells were washed in RPMI and sedimented at 300× *g* for 10 min. PBMCs were washed in PBS followed by resuspension in RPMI (Sigma Aldrich, St Louis, Missouri, USA).

### 4.2. Generation of Monocyte-Derived Macrophage

PBMCs were seeded in RPMI at a density of 1.5 × 10^6^ cells/cm^2^ and incubated at 37 °C in a humidified 5% CO_2_ incubator for 2 h. Following incubation, adhered monocytes were washed thoroughly with PBS. Cells were cultured in RPMI-1640 with L-glutamine, penicillin-streptomycin, and 2.5% (*v*/*v*) heat-inactivated autologous serum. Monocytes were differentiated to macrophage by addition of 10 ng/mL recombinant human macrophage colony stimulating factor (M-CSF) (Peprotech, London, UK) or recombinant human granulocyte-macrophage colony-stimulating factor (GM-CSF) (Peprotech) for 6 days.

### 4.3. Quantitative RT-PCR

mRNA quantification was performed using methodology and Taqman primer sets for P2 receptor subtypes, as previously described [15].

### 4.4. Immunocytochemistry

Cells were seeded on glass coverslips and fixed with 4% (*w*/*v*) paraformaldehyde for 15 min. Cells were permeabilised for 10 min with 0.25% (*v*/*v*) Triton X-100 followed by blocking with 1% (*w*/*v*) bovine serum albumin. Cells were incubated overnight with rabbit polyclonal primary antibodies (see Reference [15] for details) at 4 °C, followed by a 1 h room temperature incubation with a goat anti-rabbit Alexa Fluor 488 conjugated secondary (Abcam, Cambridge, UK). Cells were mounted with Vectashield containing 4’,6-diamino-2-phenylindole (DAPI) counterstain (Vectorlabs, Burlingame, California, USA). Images were taken using a laser-scanning Zeiss LSM510 META confocal microscope.

### 4.5. Flow Cytometry

Cells were incubated with Fc block (BD Bioscience, San Jose, California) for 10 min at room temperature prior to staining. Cells were incubated with either a mouse monoclonal anti-CD14 antibody conjugated to phycoerythrin (PE), or a mouse PE-conjugated isotype control (Biolegend). Flow cytometry was performed using a Cytoflex Instrument (Beckman Coulter, Brea, California, USA).

### 4.6. Intracellular Ca^2+^ Measurement

Assays were performed in salt-buffered saline (SBS) containing the following (mM): NaCl, 130; KCl, 5; MgCl_2_, 1.2; CaCl_2_, 1.5; D-glucose, 8; HEPES, 10; and pH 7.4. For experiments performed in the absence of extracellular Ca^2+^, CaCl_2_ was omitted from SBS and replaced with 2 mM EGTA. Macrophage resuspended in SBS were loaded with calcium indicator using 2 µM Fura-2 AM for 1 h at 37 °C. After washing in SBS, 20,000 macrophage per well were seeded in a 96-well plate and allowed to adhere for 1 h at 37 °C. Intracellular Ca^2+^ measurements were made using a Flexstation III instrument (Molecular Devices). Fluorescence emission at 510 nm was collected every 2 s, and fluorescence using excitation wavelengths of 340 and 380 nm were used to quantify changes in intracellular Ca^2+^, represented as F-ration following subtraction of baseline.

### 4.7. Drugs and Application

ATP, PSB-12062, and 5-BDBD were procured from Sigma Aldrich. U73122, MRS2500, ARC-118925, MRS2578, NF340, A438079, Ro0437626, MRS2211, and ivermectin were procured from Tocris. Receptor antagonists, inhibitors, and ivermectin were applied to cells 30 min before ATP challenge.

### 4.8. Data and Statistical Analysis

All data were analysed using Origin Pro 9.1 software (Origin Lab Corporation, USA). Data is represented as mean ± standard error of the mean (SEM). N is defined as the number of biological replicates (number of donors). Data was tested for normality using a Shapiro–Wilk test, followed by hypothesis testing by *t*-test. Concentration-response relationships were fitted using a Hill Equation, as follows:

$$Y=Start+\left(End-Start\right)\frac{X^{n}}{k^{n}+X^{n}},$$

where *k* = the Michaelis constant and *n* = the number of cooperative sites.
